# Lectures on Anatomy, Surgery, and Pathology; Including Observations on the Nature and Treatment of Local Diseases; Delivered at St. Bartholomew’s Hospital

**Published:** 1828-12

**Authors:** 


### REVIEWS.

---

ART. IV.—*Lectures on Anatomy, Surgery, and Pathology; including observations on the nature and treatment of Local Diseases: Delivered at St. Bartholomew's Hospital.* By JOHN ABERNETHY, F. R. S. Surgeon to St. Bartholomew's and Christ's Hospitals, &c. 2 vols. pp. 748. Boston, 1828.

Few names are so extensively and favourably known among our physicians (for we have no mere surgeons in America,) as that of Abernethy. Endowed with strong natural talents, practising his profession in the metropolis of our mother country, and attached to one of its most renowned hospitals, it is not remarkable, that he should erect over his transatlantic brethren, the authority which he undeniably maintains. Of the deference which is granted to his opinions at home, we are not qualified to speak; but suspect it to be less than that of the medical public in this country. This perhaps may be ascribed to the fact, that in Great Britain there are many physicians and surgeons of high domestic reputation, who have written too little to have made themselves known in foreign lands. Mr. Abernethy is not of the class of *prolific* writers, measuring him by the number of his pages, and yet besides and before the present work, he had set forth the history of his experience, and his reflections and speculations on various topics, in two octavo volumes. These, however, till lately were not circulated among us to any great extent, and it was chiefly by his treatise on injuries of the brain, on aneurisms, on tumours, on the diseases resembling syphilis, and, above all, on the constitutional origin of local diseases, that he found a passport to the confidence, we may add the admiration, of the American Faculty. The last of these works has been extensively read if not profoundly studied, and its principles and rules of practice almost as generally adopted. By a sort of acclamation, it has been pronounced a work of genius, perspicuous obvervation, and sound common sense. It is indeed the basis of Mr. A's fame in the United States. Of the merits of this popular production, it is no part of our present purpose to speak at large, but we take the opportunity to say, *en passant*, that, while we acknowledge them to be very great, they are not quite sufficient in our opinion to sustain, permanently, the elevated character which it has acquired. The chief error of this treatise is fundamental, and seems to be the offspring of unguarded generalization. The object of its distinguished author is to show, and that too from facts, that a multitude of local diseases are of constitutional origin, and require for their cure a constitutional treatment. To the term constitutional, as used in this case, exception might be taken. Chylopoietic or digestive would be a better word, for it is in a deranged state of these functions, that he finds the source of those topical maladies, and to which he applies the corrective of blue pill, rhubarb and colomba root. Now that a morbid state of the abdominal functions, as well as most of the functions of the whole body, is often associated with local diseases, is what all must admit. But is the local disease so frequently the effect of the constitutional derangement, as Mr. A. would make us believe? We apprehend that it is not; and in this consists the error of his treatise. Impairment of the digestive functions may occasion local disease in a distant part; but local diseases may on the other hand carry disorder into the nutritive organs. In the former case we must direct our first efforts upon the viscera, in the latter upon the parts affected. We know that Mr. A. admits the second member of this proposition, but we hold it certain, that he has laid too much stress upon the first. Many of our brethren moreover, have made a broader application of Mr. A's doctrine, than he ever intended, and have suffered a corresponding disappointment; but passing them by, we may appeal to his more temperate followers, for an acknowledgement, that they have frequently failed in the treatment of disorders, which on the principles of his treatise they ought to have cured. They have abridged the diet of their patients, and exhibited the mercurial pill, rhubarb and bitters, by which they have rectified disorder of the digestive functions; but the topical malady has refused to yield, and at length the constitutional has recurred. In most of these cases, the internal derangements are the sympathetic offspring of the local disease, which must be removed before they can be cured. We are aware of the re-active influence of the constitution, and that a local disease originating in the part, when it has once disturbed the general system is rendered more obstinate by that disturbance. But to expect to cure it by moderating or even subduing its distant effects, is quite a delusion. In all such cases the constitutional treatment has its limits; which scarcely extend beyond mere palliation, and if persevered in may do harm. On this subject much might be said, but we must return from our unpremeditated digression, to the new volumes, which are the immediate object of inquiry.

These volumes are composed entirely of the lectures delivered for many successive years, to the pupils in St. Bartholomew's hospital. They embrace the substance at least, of the treatises which the distinguished author had previously given to the world. Strictly speaking, they relate to physiology and pathology as applied to surgery. But in fact, they apply almost equally to medicine, and will be found nearly as valuable to the physician as the surgeon. We know of no book in the profession, that so extensively and intimately connects the two great departments of the profession. Its surgery is not operative—at least in detail; it discusses the causes, symptoms, and prevention of maladies, which suffered to continue, would render operations at last necessary; it treats of the preparation and after treatment, and discusses many of the principles on which the manipulations should be conducted;—but it leaves the rules of action to be explained by the demonstrator of the hospital, when engaged in operating before the class; and hence every physician, however averse to operative surgery, should study these lectures, and we might add, all surgical works on a similar plan. Having by this account, encouraged as we hope the bulk of our readers, many of whom are mere physicians, to enter on their perusal, we shall proceed to set forth the substance of such of them as may seem most interesting.

The introductory lecture consists chiefly of a synthetical view—anatomical and physiological—of the human body; in which the author insists, strenuously, on what no one *ought* to doubt, the necessity of cultivating anatomy and physiology as the basis of pathology. He belongs therefore to the physiological school, though he may not be quite a Broussaian. By the way, did not Mr. Abernethy's preceptor, John Hunter, long ago study the structure and functions of the body in health, as the only true preparation for the study of its diseases; and, indeed, have not hundreds of others done the same thing? How then can the disciples of Broussais justly appropriate to themselves the exclusive title of physiological?

In his physiology, Mr. A. is a true follower of his great master. He speaks of a vital principle, or subtle agent resident in the nervous system, on which the functions and phenomena of life depend. He believes mere organization incapable of producing those phenomena. Most of our readers have heard of his controversy with Mr. Lawrence, who does not travel behind the organization to seek for an explanation of the functions of life; and some of them may have seen his controversial lectures, which comprehend an *expose* of the Hunterian physiology. He evidently labours under the conviction, that this vital principle is electricity; though he seems to be rather at a loss for facts and arguments to raise the same conviction in others. At p. vii. he observes:—

"But there are other kinds of subtle matter in the nerves, such for instance, as electricity and magnetism, the motion of which does not seem to be regulated by the same laws, and which are not in general cognizable to the eye or touch, but of the existence of which reason furnishes us with the most indisputable testimony."

Now we humbly conceive, that it has not yet been *proved*, that electricity resides in the nerves, and is the principle of life, though we strongly incline to an admission of its presence and influence in all vital operations. But supposing this fluid to be the immediate agent in sensation, muscular motion, secretion, nutrition, &c., do we in that way solve the great problem of life? Certainly not. What is it that causes electricity to exert itself in the execution of the objects just enumerated? What is it that sees, and hears, and feels, and wills, when the electricity which is supposed to reside in our nerves, is put into action by external agents? It is pretty obvious that some other agent, more occult and subtle, must preside over *its* operations, and be the cause of its acting in the modes in which it is supposed to exert itself in the living body. But is that agent ultimate? The inquiry evidently tends to transcendentalism. Happily, however, we may observe and register and reason on the phenomena of living bodies, and investigate the laws which govern them, without ascending to the primitive cause of which they are perhaps remote effects.

But we pass on. Our author is not of that class of physiologists who declare, *ex cathedra*, that nothing can pass through the receptaculum chyli, but a chyle of the same quality. He is willing to open his eyes to facts; and although not a humoralist, but like Hunter, a thorough going sympathist, he allows himself to use language like the following—'As, however, many useless and probably some unsalutary particles find their way into the blood, with the chyle,' &c. This is rather an unwelcome admission, to such admirers of Mr. A. as have stoutly affirmed, in defiance of facts the most conclusive, that the chyle and blood of the same animal are, respectively, under all circumstances of the very same composition and characters. We shall leave them to deplore his heterodoxy, and conclude our notice of his introductory lecture with the following extracts, which we strongly recommend to such of our readers as have not grown rich and gray, in adapting recipies to symptoms—without the ability to analyze either.

"Now the *business* of the course will be properly arranged under three heads. The first is the *demonstrative*, in which the situation and the structure of the parts will be explained and shown. This is the most laborious part of the course, but it most particularly requires your attention. Physiology, or the reasoning on the animal functions, may be learned from books; but no books can convey to you a true idea of the shape and structure of the different parts of the body; they are so various in their organization, and so multiform in their appearance, that the true notion of them can only be acquired by the sight. I may add, that it is absolutely requisite that this part of the course should be clearly known, or the succeeding divisions of it cannot be understood.

The second part of the course is *physiological*, in which the functions of the organs, and the mode by which they perform their various offices, will be considered. The student will now be amply repaid for whatever pains he may have bestowed in acquiring a knowledge of the structure of the human body, for there is not in the whole circle of science any knowledge that brings with it so much gratification to the mind as that of the physiology of the body. While other sciences carry us abroad in search of foreign objects, in this we are engaged at home, and on concerns truly interesting—in inquiring into the means by which we 'live, and move, and have our being.' By such considerations we are most likely to form a just estimate of our own powers and consequence in the creation, and to acquire that difficult and important information, a knowledge of ourselves. But if physiology be thus engaging and instructive to the philosopher, how much more so must it be to a person employed in the practice of medicine, for he knows that it is the only way by which he can arrive at the knowledge of the nature and cure of diseases, since it is evident that the remedy for the disordered functions of the body cannot be perceived unless their natural and healthy functions be previously understood. Physiological researches are the chief means to preserve health, the most valuable of human blessings, and to prevent disease. In truth, then, I think we may say, for all these reasons, with the poet, that

'The proper study of mankind is man.'

Now the third part of the course is *pathological*, in which diseases are treated of; but this subject is too extensive to be considered in general, and therefore I confine myself, as you will see by the proposals, to such diseases as produce an alteration in the structure of bodies. The nature and treatment of injuries, which befal the bones and joints, will be dwelt on. As this subject will be most clearly understood, it will make the strongest impression on the mind, while the form of the bones, and the mechanism of the joints, are before our eyes. The operations of surgery will also be shown on dead bodies. The changes that likewise occur in the uterine regions of females during the period in which propagation is taking place, will also be a subject of inquiry and demonstration. In short, gentlemen, it is the structure, the functions of the body, the state and length of disease under the varying circumstances which take place at different periods of time, that are the subjects on which I pledge myself to instruct you, as far as my ability will permit me." —p. xviii–xx.

The first six lectures relate chiefly to irritation, inflammation, and fever. They present little of fact or opinion not already known to those who are familiar with the writings of Mr. Hunter, and other standard works. He sets out with this proposition:—

"Local disease, injury, or irritation, may affect the whole system, and induce general disorder—characterized, however, by more especial disturbance of some parts of the system.

Thus, local disease, injury, or irritation, may induce, as its immediate consequence, pain, sickness, swooning, rigors, shivering, convulsions, delirium, or disturbed state of mind, tetanus; or it may occasion various febrile affections. Now, though I think we must consider the first set of symptoms, namely, the nervous ones, as the more immediate effects of local irritation on the system, still it will be most convenient to consider the febrile affections in the first instance. These febrile affections are, some of an inflammatory character, some of a more chronic kind, and some attended with great debility. They are, in short, fevers exactly like those which Dr. Cullen has described under the heads of Synocha, Synochus, and Typhus; and no physician can distinguish them from those which occur without any external local injury. I remember the time when physicians were rather wroth that a surgeon should attempt to describe any fever at all. They were inclined to say, 'What do you know of fever? This is general inflammation which you meet with.' But I will venture to assert, that the fevers produced by local disease are the very identical fevers which physicians meet with where there is no external injury. This is a very curious circumstance, and very illustrative of medical science, as you will find hereafter; but I will not at present dwell upon it, but proceed to the description of these febrile affections. Mr. Hunter, to distinguish and appropriate them as objects of surgery, calls them symptomatic fevers, and he first treats of the symtomatic inflammatory fever."—pp. 6–7.

Thus Mr. A. is of the class who believe, that every fever is the offspring of a local affection, so that he is not remote from Broussais:—to whom, however, he should not be regarded, as indebted for the opinions he sets forth. On this controverted point, it is not our design at present, to say any thing; but we may remark, that however interesting to our curiosity it might be, to know whether every febrile commotion of the heart is the result of a local inflammation, it would not perhaps be of much practical value. We subdue the known internal phlegmasiæ of the nosologists, chiefly by means addressed to the system at large; we do the same thing in fevers, and should probably continue to do it, were it even established, that *they* also are but phlegmasiæ.

Mr. A. first proceeds to treat of what he denominates Sympathetic Inflammatory Fever, on the phenomena of which he presents nothing new. His *ratio symptomatum* is summed up in a single flippant sentence:—

> "But, hang it, I need not trouble you with this; the symptoms explain themselves; they are the natural consequences of the excitement of the heart and arteries, and the disturbed state of the digestive organs."—p. 9.

In other places, however, he speaks of the brain and nerves as involved in fever, and even seems to think that they may be first affected, which is undoubtedly the case.

"The treatment," he declares of sympathetic inflammatory fever, the symptomatic fever of authors, "is no treatment." It cannot be cured by art, it is necessary to the process of suppuration in the injured part, and we must cautiously refrain from reducing the powers of the system too low by venesection. "Your only warrant for bleeding," says he, "is that the action of the fever may perchance induce greater debility than the loss of blood." This is undoubtedly true of those inflammations which, from the nature of the injury sustained, must necessarily be cured by the secretion of pus, but it is true of them only. May not an inflammation be resolved? And for this purpose what remedy so potent as bloodletting? Every physician must have seen it *cure* inflammatory fever. The other means which our author recommends for the *palliation* of this malady, are acidulated diluents, purges, and antimonials in minute doses, which he administers, like most other practioners, to restore the suspended secretions.

When suppuration takes place, inflammatory fever is succeeded by Hectic. In his views of the production of this disease, our author follows his usual guide, Mr. Hunter. His observations on its treatment we shall extract:—

> "I will go on to speak of what is called the treatment of this fever. It is the necessary consequence of the excitement of the local malady, and if you change that, you will change the fever. What are the indications set down? It is said that you should support the strength of the patient. Aye, unquestionably, you must support his strength, for his constitution is getting weaker and weaker, in consequence of the undue action kept up by the irritation of the local disease. And how are you to support his strength? by giving him bark? This used to be the opinion, but I believe bark is less relied upon now; and for my own part I know of no mode of increasing the strength of the patient, but by increasing his powers of digestion. To me it appears that all strength arises from power of digestion, and if bark increases the power of digestion, then give it. You are not, however, to *throw in* the bark, as the phrase has been. There is a paper published in the Medical Transactions, by Doctor Heberden and Sir Everard Home, in which they state that they have never seen the bark do any good in hectic fever, unless it has been accompanied by a wound. There are wounds in which the bark is known to be an effectual remedy for a certain morbid condition of them. The bark should be given with a view of increasing the tone of the stomach, and ought not in general to be relied upon as a remedy. Tonics and cordials cannot be given in inflammatory fever, withont augmenting all the symptoms; but in the fever of which I am now speaking, they may be given in moderation with advantage. They are indeed, necessary, on account of the state of the stomach, and may be given without producing any aggravation of the febrile action. The bowels should be kept regular. The management of the stomach and bowels, however, I shall reserve as a separate subject.

With respect to the disturbed state of the nervous system which obtains in this fever, a question arises whether you may give opium to mitigate it. The answer is, Certainly. It will not do in inflammatory fever, but in hectic, where excited action is connected with irritation, opium may be given to lull the irritation, and diminish the susceptibility of the nervous system. If ever you give opium for this purpose, pay attention to the mode of administering it. Nothing is more absurd than to give a night dose of opium, which stupifies the patient and makes him sleep at night, but renders him more miserable and agitated in the day. If opium be given to diminish the susceptibility of the nervous system, in the constitutional affections arising from local disease, it should be administered regularly, in adequate doses, every six or every four hours, so that its effect may be kept up, and no intermission be suffered to take place in its sedative influence. It is necessary, at all times, in giving opium, to attend to the state of the bowels, so that costiveness may not be induced."—pp. 14–15–16.

Our author next treats of Sympathetic Irritative Fever, or that which sometimes ensues on severe injuries, in which healthy suppuration is not established, or being established, degenerates. In these cases the local and general symptoms are as follow:—

"Still keeping to the subject of compound fracture, I shall now suppose that it has gone on badly; that the wound has discharged very profusely, so that in dressing it in the morning you are obliged to sponge up the matter; that it appears flaccid and flabby; that there are no granulations; and that every thing, in short, indicates a want of energy in the constitution, to repair the mischief. The patient gets weaker and weaker, but still the surgeon cherishes a hope that a favorable change may take place, when on a sudden, the discharge from the wound, which has been so profuse, ceases altogether, or at least very little discharge remains. The wound becomes dry on the surface, and an inflammation is set up about it, which is called erysipelas. I do not think that it should be so called, but, for want of a better name, we will call it erysipelatous inflammation. Correspondlng with this change in the local affection, is an equally important change in the state of the constitutional malady. The pulse becomes strong and firm again; there is a burning sensation in the skin, and occasionally considerable perspiration; the urine is again scanty and high coloured; the tongue dry and covered with a brown fur; and there is great excitement in the nervous system, indicated by wandering of the mind, agitation, delirium, picking of the bed clothes, and subsultus tendinum."—pp. 16–17.

Practitioners of medicine in the western states are not unacquainted with this occurrence; but we have no doubt that it is much less common among them, than in the hospitals of London and other great cities. Those who are sent to such charities are too often the victims of intemperance and debauchery, while the new atmosphere which they come to breathe, unites its influence with their acquired predisposition, in producing an irritable state of the system.

"You cannot cure, you can only mitigate the disease. With this view, attention should of course be paid to the regulation of the bowels of the patient. Cordials, and any medicines that will impart strength to the patient, should be given. Opium should also be administered, to lull the general irritation, and mitigate the severity of the excitement which the local malady imparts to the system."–p. 17.

Of amputation in such a case, Mr. A. does not speak in definitive language; but it may be inferred, as the result of his experience, that the fever often continues after amputation, and that, when that operation is employed, it should be at an early period of the irritative inflammation.

In his second lecture, our author resumes the subject of constitutional irritations from local injuries, and treats particularly of the pain, the sudden debility amounting even to syncope, and other affections of the nervous system, which he justly regards as the tissue on which the various sympathies depend. When, however, in imitation of Mr. Hunter, he speaks of the stomach as the centre of sympathy, his opinion is more questionable. Indeed we confess it to be a language that we do not fully understand. Sympathy is morbid association. Now it is not the stomach, but the brain and the spinal marrow, as Whytt long ago taught us, that, by means of the nerves which re-unite in them, associate the organs together. The stomach, it is true, suffers greatly in most local irritations; but the other organs suffer likewise, in proportion to their exalted sensibilities and the functions which they perform. The whole system is *directly* injured by the topical malady, and not indirectly through the disorder of the stomach. The various organs are, in short, fellow sufferers, under the pressure of a diseased part and not of each other, or any one of the sympathizing viscera. For the syncope, or rather the tendency to it, which often takes place from a local irritation, as for example, the introduction of a bougie, the extraction of a tooth, or a slight wound, Mr. A. strongly advises the patient to be placed in a horizontal posture, and this is sound practice; but what does it prove? why clearly that the heart, connected with the irritated part by the nervous system, has suddenly become so enfeebled, as not adequately to inject the brain with blood; in consequence of which, that organ ceases to send out adequate supplies of cerebral or nervous influence, and all the functions, both animal and organic, fall into debility and disorder. Give to the heart the *hydraulic* advantage over the brain, which results from a change to the horizontal position, and diminish the expenditure of nervous power upon the muscular system, and the syncope is effectually prevented. But the stomach cannot be affected directly by change of position; and if its sympathetic affection was the cause of the impending syncope, a horizontal position could not prevent it from taking place. It signifies nothing in the argument, that a glass of whiskey and water, or a dose of hartshorn, will sometimes prevent fainting. It acts first upon the stomach, it is true, but its beneficial effects arise from the excitement which it imparts to the heart and brain. A volatile to the olfactory nerves, or cold water to the surface of the body, will often afford still prompter relief; and it is wholly gratuitous to assert that these exert their influence on the stomach. They first affect the brain, and through it the heart, the stomach, and the other organs of nutritive life, as well as animal life.

To the principle which we are discussing, our author refers traumatic Tetanus. We pass over his brief remarks on the causes and symptoms of this formidable malady, as presenting nothing new, to lay before our readers a condensed view of his experience in its treatment. With sound judgment, as a public lecturer, he generally omits all historical details, but in this instance he comes down from Hippocrates. However, Dr. Haen, Dr. Wright of Jamaica, Dr. Currie, of Liverpool, and our distinguished countryman Dr. Rush, make up the catalogue of his authorites. The Father of Physic in his opinion, comprehended all that is at present known concerning this disease. He advised the cold affusion which has sometimes succeeded and often failed. It is, in our author's opinion, like the other preservatives for this desperate accident, an empyrical practice. He knows of no specific for tetanus. The cold affusion is directed against a particular symptom; it has been tried upon horses at the Veterinary College, but gave only partial relief. He places more reliance on purgatives, and prefers calomel and jalap to salts. It is in general, necessary to repeat them for days, before free secretion takes place from the bowels, which is often productive of great relief. Mean while the sufferings of the patient should be mitigated with opium; which should be given at stated periods, both day and night, in large doses. It should not be suffered to accumulate in the stomach, and therefore cathartics are never to be omitted. That this sometimes happens is proved by a case, in which after the constant administration of a scruple by day and half a drachm by night, no less than 30 drs. were found undissolved in the patient's stomach after death.

"With respect to local treatment, suppose a man has his fingers crushed, and tetanus suddenly comes on, what is to be done? Should the finger be amputated? I see no objection to this, if the injury is such that the finger is not likely to be of any use. But will the amputation stop the tetanus? No; I think I have seen cases where it has been mitigated by amputation, but notwithstanding this mitigation the patients have died of tetanus. I would not, therefore, take off any material part of a man's body with a view of relieving tetanus."—p. 31.

To the wound, our author condemns the application of caustic and other irritants; and advises soothing applications.

At the conclusion of his lecture he informs us, that "when the irritable and sloughing state of the wound shall have gone off, when healthy granulations have formed, and a cicatrix is rapidly advancing, tetanus *may* occur." In reference to this occurrence, he proposes to the profession this question,—"*Whether the disordered state of the digestive organs, established during the irritative state of the wound, may not be the occasion of tetanus when that irritative state has ceased?*" We are not prepared to reply in the affirmative. The stomach and bowels with the other parts of the system are extremely torpid in this malady, and so they often are when no tetanus is present; something else than intestinal torpor is necessary, therefore, to its production. Our author makes no reference to the discovery of an inflamed state of the theca vertebralis, and the spinal extremities of the nerves, in some of those who have died of this malady. Such a condition offers at once a plausible explanation of the spasms and the constipation; if, indeed, the latter is not in a great degree the effect of the former, extending to the abdominal and pelvic muscles, the action of which is perhaps essential to fæcal evacuation. The same pathology will enable us to understand how it is, that in this disease the patient, to use the language of our author, is "tranquil in his mind and has his senses about him." Of cupping along the spine, a practice suggested by the theory of spinal inflammation, and which it appears has been found beneficial, Mr. A. says nothing.

In concluding this summary, which presents so little on which to rely in the cure of tetanus, we are happy to be able to state, that from some cause or causes which we do not pretend to know, it is comparatively a rare disease in the Western country. During a residence of twenty-eight years in this city, we have seen but two cases, both of which proved fatal.

Lecture third is on the influence of morbid states of the digestive organs, in producing or aggravating local diseases. Of course it involves the doctrines with which our author favoured the profession some years since, in the celebrated treatise of which we took occasion to say a few words at the beginning of this review; and we shall not, therefore, trouble our readers with a digest of that which we trust they have already studied attentively. The lecture of course embraces many exhortations to temperance, in the course of which the example of Cornaro is held up for universal imitation. We beg leave, however, to question the soundness of that philosophy, which draws a conclusion so general from a single fact. That Cornaro acquired health and comfort and long life, by changing his habits from gluttony and drinking, to rigid temperance, may be admitted; but that all men would enjoy better health and more lengthened years, by living on short allowance, is what we shall take the liberty of doubting. Many kill themselves, we grant, by gormandizing,—none, perhaps, by abstinence, except madmen, for nature prompts us in the right way. But a substantial diet is not a gluttonous diet; nor can men be made temperate in their indulgences, by declamation in praise of abstinence. The truth is, that in civilized life, where we have a great variety of functions to perform, a varied excitement of the system must be sustained, and this is to be kept up, in a great degree, by a generous diet. Mr. A. himself acknowledges that he has been a great sinner against his own rules; and still with a full stomach, he has reached the fulness of years, and a fulnesss of honors, which he would not exchange with any brother of the British metropolis. Moreover, the temperament is to be taken into account, and this presents great diversity. No general law, therefore, will bear equally on all. The sanguine and the sanguineo-bilious, may do well under rigid abstemiousness; but the phlegmatic, and they are not a few, require a liberal and stimulating diet to sustain them in activity. Dyspepsia is the penalty which our dietetic law givers attach to free living; but of this, again, we have our doubts, or rather our disbelief. At the risk of being denounced as *gourmands*, and consigned to the ranks of the heterodox in medicine, we must say, that we have seldom been able to trace up a case of dyspepsia, to excess in the *quantity* of food; and, still more, we have not been able to accomplish in its treatment, as much good by abstinence as the fashionable works of the day would lead us to expect. The bowels, in many cases of dyspepsia, require the stimulus of a considerable amount of recremental matter, and when it is withheld, they fall into greater torpor, which reacts upon the stomach. We recollect a case in point. A gentleman about 30 years of age, of the most temperate habits, had been several years dyspeptic; and the disease at length arrived at an alarming degree of violence. He took little medicine, but varied and abridged his diet in the most *exemplary* manner. For months he eat rice like a Hindoo, then drank milk as if the pastoral ages had revived, lastly took meat as if Dr. Rollo had been treating him for diabetes. But, *mirabile dictu*, in the midst of all these observances, his constipation, acidity, and emaciation remained—nay rather increased. He at length determined on a course of generous living, consisting of corn bread, bacon, beef, poultry, vegetables,—in short, the fatness of this fertile land, with a glass of whiskey and water at dinner, and very soon acquired a most comfortable *en bon point.* Twelve or fourteen years have since elapsed, and he has experienced as little of the disease, as most of his associates. Now here is a case to be put opposite to that of Cornaro. Temperate ourselves, however, we are far from intending to appear as the advocates of intemperance; and cheerfully raise our own humble testimony against attempting to cure diseases, while the patient indulges himself in a full diet or in the use of indigestibles.

The subject of the next two lectures is inflammation, and here of course our author is strictly Hunterian. The inflamed part is in a state of increased action:—It contains more blood, generates more heat, and possesses higher sensibilities. "There is a disposition in the vessels of an inflamed part to receive a greater quantity of blood than natural;" but on what this disposition depends Mr. A. does not know. The larger arteries leading to an inflamed part, as well as the capillaries, are unusually distended. The part itself is hard upon pressure, and cannot by that means be emptied of blood; which Mr. A. thinks indicates some kind of obstruction in the minute vessels, else the fluid would pass into the veins. The cause of obstruction he supposes with Dr. Cullen, to be spasm. But we suspect that in general more blood returns from a part actively inflamed, than in health. Inflamed arteries when cut, spout out blood to a greater distance than in the uninflamed state, which Mr. A. justly attributes to the vessels themselves, and not the heart, seeing that it must act on the vessels alike. The following practical remarks deserve to be remembered:—

"Here I shall call upon you to attend to a circumstance which you will frequently have to consider in practice, the hæmorrhage from diseased arteries. A patient has a diseased knee. The surgeon finds that nothing more can be done, that it has become a source of irritation to the constitution, and he advises the patient to have it removed. He is, perhaps, at the time, a little easier than usual, and he puts the operation off. He has a fresh attack of pain, and he asks the surgeon to remove it. It is done, and instead of having to secure two or three vessels, you find about twenty little vessels which bleed profusely, and perhaps requiring as many ligatures. The patient is put to bed, but the surgeon is called up in the night to stop the hœmorrhage, which has again returned, and this may happen repeatedly. This was the practice formerly, but not now, because we always amputate beyond the inflamed part."—pp. 49–50.

As to the treatment of phlegmonous inflammation, all that is necessary is to apply wet rags to the part, and then it will terminate in resolution! The great point is to carry off the superabundant heat by evaporation; but we must be careful not to reduce the temperature of the part below the natural standard. Much mischief has been done by inattention to this rule. A poultice not greased with lard, fulfils the same intention, but should not be applied cold. The *bread and water* poultice is best, and according to Mr. A. should be prepared in the following manner:—

"Put half a pint of hot water into a pint basin, add to this as much of the crumb of bread as the water will cover, then place a plate over the basin, and let it remain about ten minutes, stir the bread about in the water, or, if necessary, chop it a little with the edge of a knife, and drain off the water, by holding the knife on the top of the basin, but do not press the bread, as is usually done. Then take it out lightly, and spread it about one third of an inch thick on some soft linen, and lay it upon the part. If the part to which it is to be applied should be a wound, you may place a bit of lint dipped in oil beneath the poultice. It is very comfortable and soothing to the part. It is like a bath of tepid warm water—it produces a perspiration on the part to which it is applied. I do not know a better application. If there should be a surplus of heat in the part, you may expose the poultice, and allow the evaporation to go on a little. The poultice may be made with poppy water, if you think sedatives are necessary. It may be made also with the hemlock juice, if recently expressed, and it is a very good application to irritable sores. So also you may take a carrot poultice, but it should not be made with the great coarse substance of that vegetable. You should use the recent juice. This poultice admits of medication, but there is nothing better that I know of than the bread poultice for broken surfaces."—pp. 53–54.

If a phlegmonous inflammation is not resolved, it hurries on, with increasing violence, to a second stage—*suppuration*. The vessels in the central part of the tumour, where the inflammation is most intense, at first secrete serum—afterwards pus; while those of the circumference, pour out coagulable lymph, which cements the cellular texture; and forms a cyst. The result is an abscess, which, by an ultimate law of the animal economy, points to the surface, and when all the superincumbent parts, except the cuticle, are absorbed, that inorganic membrane is ruptured, and the pus escapes; and may be known by its containing *globules*.

"After the vessels have continued to secrete pus for a few days, the inflammation in the part, if properly treated, becomes less, and there is a gelatinous fluid poured out; into this jelly new vessels shoot, and cause little projections in the abscess; from these little projections other vessels shoot, for they soon become very vascular, and are covered with a puriform fluid. These *granulations* continue to increase, and may be seen occasionally even protruding beyond the level of the aperture. The disease has subsided, the cavity of the abscess is completely filled up, and this appears to be Nature's cure and her mode of cure."—p. 62.

The treatment of a phlegmon tending to suppuration, is with a "*greasy* poultice;" and having extracted Mr. A's recipe for making one without "*grease*," we shall transcribe his directions for the other.

"Scald your basin, by pouring a little hot water into it, then put a small quantity of finely ground linseed meal into the basin, pour a little hot water on it, and stir it round briskly until you have well incorporated them, add a little more meal and a little more water, then stir it again. Do not let any lumps remain in the basin, but stir the poultice well, and do not be sparing of your trouble. If properly made, it is so well worked together that you might throw it up to the ceiling, and it would come down again without falling in pieces; it is in fact, like a pancake. What you do next, is to take as much of it out of the basin as you may require, lay it on a piece of soft linen, let it be about a quarter of an inch thick, and so wide that it may cover the whole of the inflamed part. Then take a bit of hog's lard on the tip of your knife, and put it in the centre of your poultice, and when it begins to melt, draw the edge of the knife lightly over the surface of the poultice, and you will spread the grease over it. It is now ready to be applied, and it is what I call a *greasy* poultice. Oh! it is one of the best applications which can be made to an inflamed and irritable part, provided the linseed meal with which it is made is sufficiently fine. But I never see any such thing now as good fine linseed meal, it is all pressed into oil cake and given to cattle to fatten them.

It is, when made in this way, oh! it is beautifully smooth. It is delightfully soft. It is warm and comfortable to the feelings of the patient, and it is the best application that can be made to an inflamed part, as I have before said. The reason why I have lately preferred bread in the composition of the poultice, is, because I cannot get the linseed meal in a sufficiently fine powder. So much for the greasy poultice. You may laugh if you please, but it is a very good poultice, and the manner of making it cannot be too generally known."
—pp. 64–65.

But phlegmonic inflammation may be so severe as to destroy the vitality of the part, and occasion the coagulation of the blood in the vessels. Mortification then takes place, and how is it to be treated?

"Oh! by soothing, soothing, nothing but soothing. It is excess of action which causes the parts to perish, and therefore you must soothe them. You do not make your applications with the view of acting on the dead parts, but on the living. You must remember, that whilst the inflammatory action is so severe in the centre of an inflamed part, as to cause its vitality to be destroyed, that the neighbouring parts are still very much inflamed, and perhaps going into a suppurative process, and further from these parts the inflammation is still less severe. So that you see there are various degrees of inflammation in the parts, as they recede from the central part of the swelling. The best thing you can do, is, to apply a poultice over such a part, and attend to the regulation of the temperature of the surrounding parts, in the manner which I have before pointed out."—p. 66.

At the close of the fifth lecture, we are introduced to the subject of Chronic Inflammation,—a morbid condition of deep interest to surgeons. Chronic inflammation differs from acute, both in its symptoms and effects. It is attended with little heat or pain; the arteries leading to the part, may be enlarged, but are not affected with the convulsive action produced by acute inflammation; nor are the violent febrile commotions of the general system, which the acute phlegmasiæ awaken, experienced by the patient. The disease is indolent, and in reference to the constitution insidious and undermining. To chronic inflammation we should, according to Mr. Abernethy, attribute most of the deplorable changes of structure to which our organs are liable. A morbid action is set up, coagulable lymph slowly secreted from the blood of the inflamed vessels, into the cellular tissue, through this *nidus* new vessels shoot, the part increases in size, the natural structure is deranged, and its functions perverted. If the effusion of coagulable lymph is diffused, the proportion of new structure to the old, in any given space will in general be less, than when the secreted lymph is aggregated at a single spot. The new structure will then greatly predominate, and may even constitute the whole of the tumor; which can be detached from the normal structure, by an operation, and leave the organ from which it had sprouted out, unembarrassed. These new growths, although the offspring of a morbid action, possess an organization of their own, which may not be consistent with that of the body, or co-operative in the functions of life, but may nevertheless be so far from disturbing those functions, that the individual may continue to enjoy good health for an indefinite period of time. In other cases the sensibilities and secretions of the parasitic offspring, may react upon the surrounding parts and the system at large, in such manner as to excite general disease. On the other hand, the new structures are under the influence of the constitution; and especially, in Mr. Abernethy's opinion, of the digestive organs, the disturbances of which, not unfrequently accelerate their growth or aggravate the character of the morbid action which gave them birth. But independently of constitutional influences, they are liable to pass from bad to worse, in consequence of their own vicious temperaments or of accidental causes, acting upon them. Hence they sometimes slough off, but oftener suppurate, and the pus which they generate is vitiated and poisonous to the contiguous parts.

That the term inflammation is applicable to all the cases of morbid growth and derangement of structure, which occur in the living body, may be doubted; unless we attach to that word, other ideas than those embraced in our ordinary definitions. Many of them present none of the usual characters of inflammation, and some appear to be mere *hypertrophiæ*. Mr. A. however, regards them all as the products of chronic inflammation, and under this head proceeds in his seventh, eighth, and ninth lectures, to class and describe the various kinds of tumours—sarcomatous, encysted and purulent. As this classification has been for several years before the profession, we shall make no analysis of these lectures; but dismiss the subject with an abstract of his treatment of the inflammation, which generates them.

In the management of all tumors, it is of the greatest importance to protect them from every external irritation. They must never be subjected to great extremes of temperature, and above all, should not be rubbed or handled by the patients themselves, as is too often done. Mr. A. has known many tumors disappear from no other cause than a cessation of this practice.

It is equally important, to preserve them from the morbid effects of internal irritations; and hence the necessity, in many cases, of correcting disorders of the digestive functions, without which every topical application will prove unavailing. Of such applications, those which reduce the temperature of the part, are often more efficient than all others; but it is only the *excess* of heat that should be carried off, and we are always to avoid reducing it too low.

Local bloodletting, by means of leeches, is frequently of service; but on the whole does less good than in active inflammation. "Put on a few leeches once or twice a week, never more than six or eight at one time."

"By soothing applications," says our distinguished author, "chronic swellings may remain stationary for many months, and even years; but still there is swelling, and the object is to get rid of it by causing the absorbents to take up what has been effused." For this purpose various plans of stimulating treatment have been proposed. As an indispensable preparation for the whole of them, "all increase of morbid actions should have completely subsided," before they are adopted. So says our author. But we humbly conceive that his ideas in this respect are carried too far. An inflammation cannot always be reduced; it must sometimes be set aside by raising in the part a new action; and hence we may at the same time, in many cases, supersede a lingering morbid action and excite the function of the absorbents, by the same application. Among the means which Mr. A. advises for promoting the absorption of tumours, are frictions with the hand, bandages, stimulating liniments, electricity, blisters, tartar emetic ointment, a vinegar poultice, and issues. On the subject of electricity he has the following remarks:—

"*Electricity.*—This is a part of surgical practice which may be considered as a *unique.* All the other means which I have before mentioned, operate on the surface; but electricity will pervade the very centre of the body. It may be so managed as to be made to pervade a tumor even in the abdomen. It is a species of stimulation, and may be applied in various degrees of force. Much disputation has been raised as to the manner in which electricity operates on the nervous energy; but I shall speak of it here as a stimulant operating on a local disease. Now, with regard to its application, you must modify it according to the tenderness of the part. If you stimulate the part too much you will produce inflammation, and the tumor will increase. Now, electrify one swelling, and the swelling subsides; electrify another, and it will increase. What can we think of it? and think we must. Why we must consider that it acts as a stimulant, and increases the action of a part; that if the swelling is disposed to disperse, it disperses; and if disposed to suppurate, it goes on to suppuration. You should begin with very small charges of electricity. You would like the patient to feel it a little. Let it be managed so as to produce a feeling of warmth and tingling in the part, and let that feeling subside, if you saw that it did not increase the action too much; then touch it up again, and you might increase the force of the charge. I have frequently heard people say, if you speak to them on the subject of electricity, 'Oh! I do not think it ever does any good, I have given it a very fair trial, I was electrified every other day for two months.' All this is very possible, and they might have been electrified for two years, without any benefit, unless there be some precise mode adopted for its employment, and the effect which it produces be regarded. Now this is the worst thing that can ever be done. It is this practising without intellect that constitutes empiricism. It is blindly adopting, in this way, a power without a principle to guide it, that I abhor above all things." —pp. 74–75.

When we blister, it is not necessary to "apply a plaster a foot square, in order to derive advantage from it." Blister but a small part of the surface at once; it will then be of great benefit.

Of issues, which our experienced author prefers to setons, he speaks in terms of commendation which we shall extract:

"*Issues*—I hold to be the most lenient, and the most effective counter-irritant; and when I have to keep up this counter-irritation for a long time, I generally make an issue. What I say is, let me have a chronic remedy for a chronic disease. You may make a sore upon the skin with a hot iron if you like, and they say that it is the most lenient way, if it be hot enough; and a good deal has been said in its commendation. But there is something so formidable in this mode of producing an ulcer, that it would completely terrify some persons. I do not like it, and I never did like it. Now, if I had to make a sore upon the skin, with the intention of keeping that sore open as an issue, I should make it with a caustic, but not such a caustic as would spread all round the place on which it was originally applied, and produce a sore twice as large as I intended. This would be just as bad as the hot iron, in my opinion. Now I like the old caustic best; that is, the caustic made with the potash and quick lime, for with it you may destroy just as much of the skin as you like, and no more. This caustic might be applied with so much precision, that I would engage to sketch out upon the skin any fantastical figure that you would tell me; aye, even if it were such a one as George and the Dragon. The way in which this is to be applied, is the following:—You take a piece of adhesive plaster, and cut a hole in it of the size that you wish the caustic to be applied. You put this on the skin, aud over it put another piece of plaster of the same shape. You go on putting one piece over the other, until you have formed a cavity in the centre to hold the caustic. You then take a piece of the caustic I have last mentioned, wet it a little, put it into the hollow on the skin, and over it put another piece of plaster. The patient says, 'Oh! dear, how nice and cold it feels!' You let it remain there about four hours and a half, or five hours, and before that time he finds out that it is not quite so comfortable. You take it off, and you find that there is a good eschar produced. The skin is not a black eschar, like what it is after applying the other caustic, but nearly of the natural appearance. This comes away in a very few days, and you have a wound produced of exactly the dimensions you intended. If you wish it to discharge much, you may dress it with some stimulating application. Peas are commonly used for this purpose, but I considered that there was great inconvenience attending the daily changing of the peas, and was induced to substitute some glass beads. The wound can only heal from the edges, and if you place any foreign body there, you prevent the process of cicatrization as entirely as if you were to cover the whole surface of the wound. What I have recommended, is a string of small glass beads to be placed round the margin of the circular sore, which become in a few days buried in the granulations, and give no further trouble. Mr. Brodie has said, that the best plan is to touch the sore over, when necessary, with a little pure potash."—pp. 77–78.

The 10th lecture opens with some brief remarks on Irritative Inflammation, by which our author means that which attacks surfaces and is prone to terminate in *mortification*. It is sometimes *located*, to use a phrase of the Backwoods, in the absorbent glands. It often intermits. Not limited to the skin, it is frequently established in the walls of abscesses, and in the surfaces of wounds and ulcers. In every case it is attended with constitutional irritation, and a fever of the irritative kind. This inflammation has been called erysipelatous, but Mr. A. doubts the propriety of the denomination; and dismissing the subject without favouring us with his *methodus medendi*, he proceeds to speak of the latter affection.

Erysipelas is a cutaneous "travelling inflammation." It generally spreads by continuity of surface, but sometimes leaves one spot and appears on another. Not unfrequently, it suddenly disappears from the surface, especially under the influence of certain applications, and the heart or brain or some other vital organ, becomes seriously, even fatally, affected; but according to the observations of Mr. A. it is not erysipelatous. Their structure does not admit of that inflammation, and what their pathological condition is, he does not know. Erysipelas is not apt to terminate in mortification, like irritative inflammation: whether it ever suppurates, our author is not prepared to say. Every physician, however, must have met with cases in which this inflammation was connected with suppurative inflammation, in the subjacent cellular tissue. Erysipelas may come on from constitutional causes, or from local injuries. Mr. Abernethy's pathology and treatment of the former variety, is summed up in the following paragraph:—

"The occurrence of erysipelas shows something very wrong about a man's health. Let something be applied to provoke a part a little, let there be an increased action produced, and in a healthy man you will have a phlegmon; you will have none of this sneaking inflammation, which I am now telling you about. The general health was disturbed before the functions of the digestive organs were disordered. The person has nausea, and loss of appetite, and the actions of the different parts are not healthy;—and that settles it. It is produced by the state of the general health, and it acts upon the general health. I should put a little bread and water poultice on the part, and try to soothe the disturbance which has been produced."—p. 121.

For the production of surgical erysipelas, a wound alone is not sufficient; there must be disorder of the digestive organs. This opinion is clearly expressed by our author in a single eloquent sentence:—

"I'll be hanged if erysipelas is not always the result of a disordered state of the digestive organs. I never see it come on if the digestive organs be right, and it goes away as soon as they are right."—p. 122.

In the management of these cases, half a grain of calomel and five grains of jalap may be given every four hours. Cold or repellent applications are to be avoided. Of blisters, mercurial ointment, and other strong measures, Mr. A. says nothing.

The lecture concludes with a pathological notice of the FURUNCULUS or boil; and the 11th begins with a short account of the CARBUNCLE, which Mr. A. thinks generally arises from some disorder in the digestive organs. Let him speak for himself:—

"I have no doubt that the cause of the complaint is a disordered state of the digestive organs, and of the mode of treating that disorder I have already spoken. If I could set all the digestive organs to rights; if, I say, I could set this disorder to rights, egad! I should do better than if I discovered a philosopher's stone, and had the knowledge of the art of prolonging life."—p. 129.

Carbuncles should be opened with a free incision, by a double edged knife.

The ANTHRAX Mr. A. believes also arises from digestive derangement, and advises it to be treated in the same way with the carbuncle.

MORTIFICATION comes next into notice. "It is the want of life in a part." "It is a vital process." "It is the last act of life." We do not, however, perceive very clearly, how the want of life can be an act of life! A great variety of causes may induce this state; and it, therefore, requires a variety in its treatment. For the mortification from cold, cold applications are necessary. The transition to a higher temperature must be gradual. When the disease arises from weakness, it is generally associated with disease of the digestive organs and an irritable state of the whole system. If it arises from excess of action, we must not stimulate, generally or topically, but apply a bread poultice.

"The forms of disease which I have been mentioning this evening, may be traced to that state of health, which, in the early part of these Lectures, I mentioned as being the fruitful parent of a very numerous and dissimilar progeny of local diseases; a state of health produced through the reciprocal action of the digestive organs on the nervous system, and the nervous system on the digestive organs." —p. 137.

The mortification of the toes and feet in old people, is generally dependent on an ossification of the small arteries:—

"In the treatment of mortification of the toes, it is wrong to apply stimulating applications. What I usually give as a medicine in these cases, is the camphor mixture, with a little volatile alkali; or, if you please, you may give a little cardaic confection. Mr. Pott has written about the advantage of opium in this affection. If you find that there should be much pain, then opium will give great relief, but unless there should be much pain, I do not see what advantage is to be gained by it. There are many instances of mortification having taken place without any pain being felt."—p. 138.

Such in substance are Mr. A's views of the nature and treatment of mortification. They are certainly not as full as might have been expected; but we pass on to the next lecture.

The first topic of this lecture is Œdema, which our author thinks is owing to increased anterial action, oftener than to diminished absorption. In Elephantiasis there is a deposition of gelatinous matter. Of the treatment of Œdema:—

"As it is probable that in general there is an increased deposition, we must try to check it, and to cause absorption. Upon my life, I know of nothing better than bandaging and gentle friction, and attention to the general health. Pressure promotes absorbtion, it supports the weak vessels."—p. 140.

This leads our author to speak at large on the subject of absorption, a process to which he attributes the progress through the body, of pins, needles, &c. when they happen to be swallowed:—

"A gentleman, who was a student here, sent me a case of that kind, in which, by some accident, for I may say it was a mere accident, the woman knew how many pins she put into her mouth. I forget now exactly the number, but it was between forty and sixty. They all came out in about six months, from different parts of the body, shoulder, breast, belly, legs, arms, and back. During the whole of this time, she had most horrible nervous irritation. At last they were all discharged and she got very well."—pp. 141–142.

But the passage of foreign substances through the body, is not always attended with nervous irritation.

Interstitial absorption leads to the formation of ULCERS; maladies of such frequent occurrence, that we shall present in a long extract the remarks of our author on their treatment:—

" *Treatment of Ulcers.*—First, of the treatment of *common ulcers*, ulcers in which there is no morbid propensities. I have told you as much as I know as to the principle on which all ulcers should be treated. The irritable sore must be soothed, and the indolent sore excited.

There is nothing that can be applied to a sore, when in a quiet state, so good as Baynton's bandage. First, you appease the part, then you apply a slight stimulus, such as the sore may tolerate without resentment, without exciting an inflammatory action; and this is what I call the surgery of *susceptible surfaces.* You diminish sensibility by exciting the part to a certain degree, short of that which produces reaction. Now I never can explain my meaning half so well as by referring you to a fact of which you must be peculiarly conscious; I refer to an inflamed eye. What is necessary to be attended to in the case of an inflamed eye, is necessary to be observed in the manner of applying Baynton's bandage, if you expect it to be attended with success.

Well, what do you do when a man's eye is inflamed? You tell him to apply leeches, to use cold washes, or the solution of opium, to take aperient medicine, and so on; you do every thing, in fact, to quiet and tranquilize the part, to allay the inflammatory action, and when you have succeeded in doing this, you adopt a different plan. You tell him to use an eyewater, and you give him a weak solution of the sulphate of zinc, say one grain to the ounce, and you tell him to use it frequently, to use it every six hours, to apply it by the means of the eye-cup or by putting a little of it into the eye. The first time he applies it, he puts his finger to the eye and rubs it, for he finds that it smarts uncommonly; he probably turns about, stamps on the floor, raves from the violence of the pain, and is almost disposed to damn the doctor who puts him to this, as he imagines, unnecessary pain. The pain, however, soon goes away, and he is induced, from his eye still continuing weak, to use it again. He does so, and finds that it smarts much less than it did the first time; he tries it again, and finds that it smarts still less. He says to himself, 'Hang it, did not the doctor tell me to use it every six hours? I will try it again.' And so he goes on till he finds it makes no more impression on his eye than water by itself.

The strength of the collyrium is increased, he uses it without producing any serious inconvenience, and the eye is cured. Now, I ask you, if this does not show a morbid susceptibility of the surface to which the stuff is applied, and this morbid susceptibility is removed by the application of a stimulus not sufficient to produce reaction or any sort of inflammatory process.

There is a great necessity for applying stimulants to sores, but the time for using them must be regulated by the condition of the sore. If you apply a stimulant after you have excited the irrtiability of a sore, granulations are formed, which continue to grow; but if you increase your stimulants the granulations will slough. But while the sore is ındolent it will bear the application of a stimulant. Now, as to the manner in which Baynton's sticking plaster bandage should be applied, and the good effects which it is capable of producing, they are both parts worthy of attention. It is ridiculous to suppose that there was any peculiar benenfit to be derived from its being applied by any particular individual, for what can be done by one can also be done by another.

It is a bandage made of straps of adhesive plaster put carefully round the limb, having the sore first protected by a little lint dipped in a solution of opium, or by a bit of lint having a little spermaceti salve spread on it. The straps of plaster are put round the lower part of the limb first, and then made gradually to come upwards. Over the whole you may pass a light roller. Great attention is necessary as to the manner in which it should be applied, and I wish to call the attention of the dressers of this hospital particularly to this subject, for unless it is done well, it cannot produce those marvellous effects of which it is capable. I recollect the case of a clergyman which very well illustrates its utility. He called on me and said, that he entertained a great respect for me, and wished very much for my opinion respecting his leg. He showed me his leg, and there were two sores on it, which he said had been very intractable, and that he had for many months felt great inconvenience and pain from their continuance. I said, 'Oh! if they are dressed properly, they will be well in a fornight.' 'A fortnight! surely, sir, you are in jest, I have been under the management of some of the most eminent surgeons in London for many months, and now you say I may be well in a fortnight!' He appeared very much hurt at what I had said to him. 'Nay, sir,' said I, 'you need not be wroth at what I told you. I saw that you were exceedingly anxious, that your mind was in an irritable state, and I said it to pacify and tranquilize you, and I again repeat, that if your leg be properly dressed, it will be well in a fortnight.' He said that if that were the case, although he had very important business to take him out of town, he would remain for that time at least. I ordered him merely a dose or two of blue pill, and a little aperient medicine, applied Baynton's bandage, advised him to keep at home and raise the ıeg in a horizontal position. And before the fortnight was over, aye, even before five days were over, his leg was quite well.

I remember also the case of a young lady, who had a bad ancle. It was near Christmas time, and she wished very much to join in a dance with her other sisters, who were to go to a ball in a week. I said that a week was a short time to heal a sore in, but, however, we should try what could be done. I put on the bandage I have just been speaking of, and she went to the ball at the end of the time I have mentioned, and danced without any inconvenience.

It appears to me that Mr. Baynton did not, at first, exactly understand the principles on which this bandage of his was to act. He imagined that the benefit was obtained by its approximating the edges of the sores. But I think it acts differently. It acts by giving a moderate and equable support to the newly formed vessels of the sores, and supplies the deficiency of the proper natural coverings. I remember a man in this hospital who had a large ulcer on his leg. He could not even put his foot out of bed without producing most excruciating pain. He had the bandage applied, and was desired to stand up by the side of his bed. He did so without making any complaint. The vessels of the leg had received the support which their weakened state required, and therefore were not overdistended as before.

Then again, if it should be necessary to lessen the temperature of the limb, you can do this very easily by wetting the bandage with water, and allowing it to evaporate. Mr. Baynton was aware of this, and he recommended the patients, when the heat of the limb became high, to sprinkle water over the bandages, and to do this there is nothing better than a watering pot. The bandage retains as much of the fluid as is necessary for the process of evaporation to take place. It is a very useful, and, generally, a successful method of treating sore legs.

But to return to the various methods which have been proposed, and the various stimulants which have been recommended for the treatment of ulcers. It is impossible to describe all the appearances which present themselves in the progress of ulcers, or to detail to you the stimulants that are applicable to particular cases. The practice in these respects is rather empirical, for we are guided principally by experience. Descriptions cannot convey to you correct ideas on these points; you must see them to understand them.

I said there were various methods of stimulation practised, and sometimes with very good effect, but I am satisfied that I have seen ulcers get well simply by attending to the constitutional treatment. A man in the hospital had a large sore on his leg for a very long time. It would improve under a new stimulant for a short time, and then require another. I said one day, when going round the house, 'I wish you would dress this man's leg with a little simple ointment, merely attend to the state of his digestive organs, and give him a little sarsaparilla.' They did so, and he got speedily well.

I have said that if a man would give me a Pharmacopœia, I would undertake to say that there should not be one thing contained in it, which, sooner or later, I have not seen applied as dressings to sores. All the substances which it contains may be ranked under particular classes, and their application to ulcers may at one time or another be useful.

There are substances which may be regarded as stimulants—the different preparations of the metals, of mercury, zinc, copper, and so on. The mineral acids may be ranked under this head, and they certainly, when diluted, are very useful applications, as the nitric or muriatic acids. I once took it into my head to try the effect of bathing the limb in an acid, where there was a diseased bone. I put some nitric acid into a tub of water, and told the man to soak his leg for a quarter of an hour in it, for the purpose of dissolving the earthly matter of the bone, but I found it produced a great deal of inflammation, and I was obliged to give it up. Then again, there are substances which may be called tonics. These, when applied to sores, often produce good effects; such as the astringent vegetable barks, and so on; other substances which may be called cordials, as the different balsams; the gum Benjamin, the solution of it, called Turlington's drops, and to some indolent sores it is a very good application. Other things act as sedatives or narcotics, and you know there is a very large variety of substances that act in this way. There are opium, hemlock, and belladonna, and so on. As to the latter substance, I once had a case in this hospital where the sore had been very irritable and obstinate for a long time, and we had tried almost every application without any benefit, when I recommended him to use an ointment, in which some belladona had been rubbed down. He did so, and passed a very quiet night; he was quite delighted when I saw him in the morning. Well, I thought this was a very good application, but just as I was going away from his bed, he said 'Do you know, sir, that I had a very odd thing happened to me this morning? I was dizzy, and could not see any thing for an hour.' 'Oh! were you?' said I. But, egad, I did not think then it was a very good application, and I ordered him to leave it off immediately.

Having now described those ulcers, which may be called simple, from their not having any morbid peculiarity, such as the indolent ulcers, which Sir Everard Home has described, and which he says bark will cure; such as the irritable ulcer, for which he recommends mercurial ointment; there are other ulcers, he says, which a solution of common sea salt will cure—these I take it for granted are the scrofulous ulcers—I shall next speak of ulcers which are characterized by having some *morbid peculiarity*.

The cancerous ulcer is of this description. It is an ulceration attended with violent pain, the discharge from which is horribly fetid, and it is a very intractable disease. Different applications have been made to such ulcers with a view of correcting the nature of the discharge, and allaying the irritability of the sore; the oxides of the various metals, carbonic acid gas evolved from fermenting poultices, weak solutions of the oxy-muriatic acid, Chlorine, as it has been lately called, but I do not know what they call it now—they change the names of things so often.

The discharges from such sores have been made the subject of analysis by Dr. Crawford. He found that this ichorous irritating fluid contained sulphurated hydrogen gas. It is owing to its containing this, in combination with other things, that the common adhesive plaster, containing lead in its composition, becomes blackened. It is sufficiently powerful, very frequently, even to blacken silver probes that you may use in dressing. I know of nothing better for the correction of such discharges than fresh burnt charcoal. There was a druggist, a scientific sort of man, and he had a jar of water, in which some leeches had been kept, and in which many of them died, and the water became very putrid. He wished to ascertain what could be accomplished by the charcoal, in changing the appearance and quality of the water. He burnt some wood, powdered it almost directly, and put it into the water. In a short time it became clear, and was deprived of its putrescent matter. It appears to me, that it is the best application that can be used to such sores, joined to the hemlock poultice. You must quiet the irritation of the system as much as you can, and give anodynes, and so on, and attend to the state of the digestive organs."—pp. 151-157.

Lecture 14th opens with VARICOSE ULCERS. Our author condemns the ligature to varicose veins, as proposed by Sir Everard Home, on account of the inflammation of the vein and the constitutional irritation which it excites. Whether Mr. Brodie's operation with the knife is safer, he is unable to say. The nervous irritation which sometimes follow the ligature, suggests to his mind the subject of nervous affections generally, and he devotes a couple of pages to the influence of these affections upon the circulatory and absorbent functions. "Every disease is originally nervous. Disorder of the nerves is first excited, and this leads to increased action of the vessels, and increased action of the absorbents of the part." "Some diseases affect the vitality of the part." "There is a certain disorder of the nerves of a part antecedent to disease in the part. Suppose that there is disturbance in any part, you can not say that it is disease; it begins with irritation, and therefore I object to the word disease in that sense." In this language there is a singular degree of looseness. Our author's ideas are surgical to excess. To him the word disease evidently conveys the idea of a *sensible* change of structure. But disease is merely disordered function, and that irritation of the nervous function of a part, which precedes and produces disordered circulation and absorption, is as much entitled to the epithet diseased, as are the aggregated disturbances of the three great tissues which he enumerates. When he says that *some* diseases affect the vitality of the part, his language is still more exceptionable.

The lecture concludes with our author's opinions on Tic Douloureux. This most painful and obstinate malady, he asserts, is far more common at the present time, than in the first period of his professional life. He regards it as strictly constitutional, or rather digestive, in its origin. The excision of the affected nerves he condemns us useless, and resolves the treatment, into a proper rectification of the disordered digestive functions, by means of diet and suitable prescriptions; but on the whole he regards the disease as being generally incurable.

From Tic Douloureux he proceeds by an association of ideas (as obscure as the condition of the system in that malady) to treat of Diseases of the skin. At the outset he objects to the formidable attempts of some modern nosologists to subject them to an elaborate classification. The new names which have been introduced "bother" him; and he is certainly far from being the only one whom they have perplexed; if not driven from the subject.

"There are causes which act on the exterior of the body, as cold and wet, which weaken the circulation of the skin, that may lead to cutaneous disease; and many medical men, in order to keep up a certain degree of energy in the action of the skin, employ tonics in cutaneous diseases. The decoction of elm bark is one very much used. But I think the great cause of all variation in the states of the skin is to be met with in the digestive organs. Cuvier, speaking of the alimentary canal, says, that it is an internal sac of the skin, that the skin is inflected and lines the intestinal canal. You may have perceived that diseases of the skin arise in general from irritation of the alimentary canal, making allowance that weakness and defective circulation may sometimes be the cause of them. The main cause, however, is to be found in the state of the digestive organs.

The great means of curing cutaneous diseases is by tranquilzing the state of the alimentary canal. I have continually observed, in patients with these affections, that as the digestive organs were set to rights, so the cutaneous diseases were cured."—165-166.

When local applications are resorted to in diseases of the skin, if they be oleaginous, the parts must be well washed and dried before they are put on.

"The best medicated pomatum, for so I call it, is hog's lard, rubbed down with the liquor plumbi subacetatis—this is the name of it I believe—I mean Goulard's extract, in the proportion of one drachm of the latter to an ounce of lard. There are many diseases of the skin in which the unguentum hydrargyri nitratis is of use. It should not be applied too sharp—two thirds or three fourths of lard, to one third or one fourth of the ointment. I have seen many cutaneous diseases cured by the unguentum hydrargyri nitratis. Tar ointment and tar water are also good applications. This leads me to speak of washes, which produce certain good effects in cutaneous irritation. A solution of sulphate of zinc and of Goulard's extract are good washes. Also, water impregnated with sulphuretted hydrogen, by a solution of sulphuret of potass, formerly called hepar sulphuris, and the tar water, frequently do great good."—p. 168.

Many diseases of the skin produce ulceration. For PORRIGO our author suggests no application. Mercury given so as to salivate, will not cure it, but may do good, by correcting disorder of the digestive organs. Of HERPES he speaks in nearly the same language. For PSORA he advises brimstone and lard; and for TINEA, the white precipitate ointment, shaving the head, and due attention to the state of the digestive organs.

In the first part of his 15th lecture, our author speaks of inflammation of the absorbent vessels and glands; which he insists has not been sufficiently considered, by the profession. It is, in his opinion, a pathological state of frequent occurrence. It is often the result of external accidents—the constitution at the time being unhealthy. The dangerous inflammation, with irritative fever, occasionally following on DISSECTION WOUNDS, he believes to be owing to the state of the constitution, and not to the impress or absorption of a morbid poison. His remedies are a bread and water poultice, rest, and correctives for the digestive functions. To SCROPHULA he devotes but a couple of pages, and refusing all confidence to specifics, looks only to the improvement of the general health, by the common means.

He now comes to diseases from external violence, and other extraneous causes. Bruises and wounds first present themselves, and he concludes the lecture with a brief account of Mr. Hunter's experiments on adhesive inflammation, which we need not analyse.

The succeeding lecture contains his opinions on the treatment of wounds, on hæmorrhage from wounds, and on hæmorrhage from the surface of ulcers. In the whole, we see nothing new, except perhaps that the latter bleeding is generally the consequence of a disordered state of the digestive organs, and requires to be treated with cathartics and other constitutional remedies. When speaking of adhesive inflammation, he refers to the restoration of noses and lips by the Taliacotian operation. Of the former he has never seen one equal to those made of *paste board!* but of the latter he seems to entertain a more favorable opinion; and as the operation appears to be one of greater practicability, we shall extract all that he says concerning it:—

"A new lip may be formed from the skin under the chin, and really this is a very important and useful substitution, both in the process of mastication and for the purposes of speech. A gentleman of this town cut out a piece of the integument from beneath the chin, just of the size he thought would do for the lip. He fitted it very nicely into a grooved edge which he had cut to receive it, twisted it about, and it united perfectly, although it was a long time before the sensation returned in the part."—p. 185.

The remainder of the lecture is on Fractures. In the management of these casualties, Mr. A. is a decided opponent to "extension, counter-extension, coaptation, delegation, and so on, and of the various powers to be used for the accomplishment of all this nonsense."

"The manner in which I should treat a fracture would be just this: I should have the patient laid in bed, and the limb, resting on a splint, should be placed in that situation in which it might remain for any length of time. I should make a gentle effort or two to replace the limb, or at least to bring it as near as I could, into the natural position without much effort. If I found the muscles pulling obstinately,

I would let it alone, place the limb on a splint, and then on a pillow, so that it may rest easily. I should, in addition, just give a little something to open the bowels. The resistance made by the muscles is, I say, seldom longer than the period of twenty-four hours, and then you may manage it as you please. When once you have got the limb into its proper shape, the muscles will preserve it as pertinaciously in that position as they before resisted your attempts at doing so. Nay, sometimes they even assist in bringing the fractured ends of the bone in their proper situations."—pp. 194–195.

The period at which fractures are united, varies exceedingly in children and adults, and even among persons of the same age. The patient, in general, has a feeling of "animal consciousness" which enables him to decide whether the bones have united.

Fractures sometimes refuse to unite. This, in our author's opinion, is owing to different causes—a bad state of health in the digestive organs, motion at the time when ossific action is taking place, or sufficient time not being allowed. His remedy is protracted rest, and the improvement of the general health. Sawing off the ends of the bones often fails; and the introduction of a seton between them, as practised by our distinguished countryman Dr. Physick, designated by Mr. A. as "a gentleman in America," is still more unsuccessful. Our author has seen it produce great constitutional disturbance. Of tight bandaging as recommended by Mr. Brodie of London, and Dr. Wright of Baltimore, he says nothing.

The difference between a Compound Fracture and a simple fracture is that the latter "heals by adhesion, the former by granulation." If the blood extravasated about and between the fractured ends, can be kept from escaping, new vessels will shoot into it, bony matter be deposited, and the cure accomplished as in simple fracture; but if it escape, the cavity which it occupied must fill up with granulations. To effect this, Mr. Mudge closed and secured the wound very accurately with strips of adhesive plaster, which he varnished over, so as to make a covering not unlike the integuments; and this has often succeeded. In cases of this kind Professor Dudley, see our respected Western cotemporary, the Transylvania Journal, directs a tight bandage to the whole limb, so as to paralyse the action of the muscles, and prevent inflammation. The indication of Mr. M. must at the same time be fulfilled. In compound fractures Mr. A. adds:—

"You may very much lessen the inflammation, and prevent the suppurative inflammation, by diminishing the temperature of the part. There is no measure so useful in the treatment of inflammation, as that of the regulation of temperature. Lay thin cloths, wetted with Goulard's lotion, or spirit and water, over the part, to diminish any excessive heat that may accumulate there, and you will do a great deal towards preventing the evils frequently attendant on a compound fracture. Keep the parts at the same time on a splint, and perfectly quiet. It is a point of very great importance in the treatment of fractures, to place the limb, and to place the patient, in that position from which there may be no necessity to remove him for a considerable time."—pp. 205–206.

In compound fractures attended with hæmorrhage, Mr. A. has found cold applications to the wound quite sufficient. For the same purpose Professor Dudley, *loco citato*, relies upon the bandage. The two methods might be united by those who believe in the *safety* of the roller.

When the bones project through the integuments in oblique compound fractures, our author, following Mr. Pott, prefers enlarging the wound, for the purpose of effecting their return, to sawing off the protruded ends, which of necessity lengthens the space to be filled with granulations.

In comminuted compound fractures, our author advises that as many as possible of the fragments should be replaced, or left *in situ*. In accidents of this kind, requiring amputation, he does not agree with Mr. Guthrie that it should be resorted to, immediately. He would wait until the system had recovered from the shock. On the question what kind of comminuted compound fractures required amputation, we shall let our author speak for himself:—

"It does not follow, as a matter of necessity, that because a bone is comminuted, and the soft parts contused, that you must operate. There are many such cases that do well. What then are the principles which ought to guide you in determining on the operation? It is quite impossible to lay down any precise rule, founded on this or that appearance of the parts; or to say, that because this artery is torn, or that part is injured, or that the bone is broken in this or that manner, that you must operate. But if there appears to you to be such mechanical injury done to the living structure as could not be ultimately repaired by Nature's processes, or, that if reparable, would, from the state of the person's general health, make such demands on the vital powers as they could badly support, then you might be justified in amputating. Rules! there is but one rule in surgery, or there ought only to be one rule—*Do unto others as you would have done to yourself*. If you can say, after asking yourself, if your limb were in the condition of that man's, you would have it removed, then I say, you can with great propriety recommend it."—pp. 211–212.

In the 18th lecture, Mr. A. delivers the results of his experience on Burns and Scalds. He is an advocate of the Kentish plan, and after telling his pupils in what it consists, bears to it the following testimony:—

"I deem this to be a rational mode of procedure, and I think it has been condemned because it has not been properly understood. Recollect, that this stimulating plan of treatment is not to be continued after the equilibrium of the temperature is restored, and after the patient has a degree of heat and reaction established; that was never intended; you are then to dress the scald with mild applications, and leave off giving the patient cordials. He may then require a little aperient medicine, and so on. Neither are you to put your stimulating applications on a surface which has been denuded of its cuticle. Where the cutis is bare even the mildest application to that will give great pain. You should in that case give the patient a small quantity of opium in a little cordial, and put a little simple dressing on the sore. Dr. Kentish says that he has found finely powdered chalk, with which a small quantity of opium has been mixed, the best dressing for such scalds. It appears to allay the irritability and promote the cicatrization of the sore.

But supposing a scalded part to be followed by an inflammatory action and sloughing, there is oftentimes a great demand made upon the vital powers of the system to separate the slough, and even the energies of the parts surrounding the slough become fatigued. They are in that state which Dr. Brown calls *indirect debility;* and in such cases, a gradual support to the system will be necessary. This is one of the most delicate points to manage in the treatment of scalds and their consequences; the degree of support necessary to afford the system, without the danger of bringing about too much reaction. There is a very great sympathy between the surface of the body and the stomach. When a person has an extensive injury of the skin, he is, as I before said, chilly, and has a feeble pulse, but he has also vomiting very frequently; the stomach is evidently disturbed. The digestive organs suffer, and Dr. Kentish has recorded a very notable fact to this effect. He observed that many of the injuries of the skin cicatrized rapidly, when a spontaneous purging came on, and this led him to prescribe, in some cases, aperient medicines, with a view to facilitate the healing process, and he says that he succeeded very frequently in accomplishing it.

I can speak from experience and say, that the mode which I have now mentioned of treating scalds is a successful method, if properly managed. We have had a great many such injuries in this hospital, and since this plan has been pursued, they have generally done very well."—216-217.

From burns and scalds our author proceeds to MORBID POISONS; and this leads him to consider syphilis, gonorrhœa, pseudo-syphilis, and other diseases of the bladder and urethra; but as his views relative to most of these subjects have long been before the profession, we shall pass them over, with a notice of the disorder termed IRRITABLE BLADDER.

"The bladder is often irritable, capricious in its actions, and tender in its lining membrane, so that it will not bear distention; capricious in its actions, acting partially, not emptying itself entirely, and therefore liable soon to become distended again; not bearing distention, and tender in its lining membrane, and on that account irritable. The lining of the bladder may go on to ulceration, there may be a puriform secretion, and blood may be discharged with the urine; yet all these cases go under the title of irritable bladder. I have known blood and pus discharged in the urine without ulceration; but when the bladder does go into a state of ulceration there is but little hope of recovery. The function of the bladder is such as almost to preclude the possibility of an ulcer healing. Acid urine is continually applied to the sores, the ulcer is stretched by the distention, and even injured by the contraction of the bladder. You can hardly expect an ulcer of the bladder to get well, and, generally speaking, it is a lost case. I know not what is to be done. There are no remedies, but those of steadily pursuing the plan of soothing the local irritation, and putting the digestive organs to rights; and I have known instances in which people have got well, which you will hardly credit."—p. 303.

Mr. A. knows of no specific for an irritable bladder. The uva ursi has been recommended, but it is only of service "when there is a little discharge of mucous from the bladder, with a degree of languor of the patient's constitution." Mild doses of the carbonated alkalies do good; but in large portions, they increase the irritation. He has known the balsam copaiva and oil of turpentine to be useful, but they often fail; and his chief reliance is on reduced diet, and a proper rectification of the digestive functions.

Returning from a long digression, he resumes the subject of morbid poisons, to speak of that which produces HYDROPHOBIA. This virus differs from most others in affecting all kinds of animals; but whether they all generate it, we do not know, as many of them are not biting animals. Cats can propagate the disease, but Mr. A. thinks that it never arises, spontaneously, in them. He indeed doubts whether it ever thus arises in dogs. This poison differs from many others also, in this, that it does not commonly inflame the wound, which heals without difficulty. For hydrophobia, when it is established in the system, Mr. A. knows of no remedy. In the treatment of the bitten part, he prefers excision to the caustic, or cauterization, and considers the latter useless, if the former has been properly performed; that is to say, in such manner as to remove the whole of the wounded surface. He has, however, known the disease to occur after an early and adequate excision had been practised. Before operating, the wound should be washed with soap and water.

His 26th lecture embraces diseases of the LACHRYMAL DUCT, and of the ANTRUM HIGHMORIANUM, with the FURGI which it occasionally shoots out. For these affections he recommends the usual operations, which we need not describe.

A similar remark would apply to the subject, which makes up the first part of the next and last lecture of the volume. It is dislocations, fractures, and diseases of the lower jaw, on which we see nothing of particular interest to extract, and shall conclude our analysis of the volume, with an abstract of our author's observations on DISEASES of the SPINE.

Mr. A. believes that there is such a thing as CONCUSSION of the SPINAL MARROW without dislocation or fracture of the vertebræ, but it is extremely rare. DISLOCATIONS he thinks scarcely ever happen, without a fracture of the processes or body of the bone. In these cases the projecting bone is *in situ*, the other is separated from it. Is it safe to employ extension and counter-extension in these accidents? Our author thinks it dangerous to the spinal marrow. The proper treatment is rest, perfect rest, in a horizontal posture; with the appropriate means of preventing inflammation; and at a subsequent period, stimulation of the limbs with frictions and electricity.

The spine is subject to CURVATURES. Many of these are mere deformities, unaccompanied by any diseased action. They result from tight lacing, constrained positions and movements, some weakness or defect in one of the legs, by which the weight, and consequently the specific gravity of the body are thrown chiefly over the other, &c. But an unhealthy state of the constitution is generally present. In the treatment of this affection, our author condemns the use of machinery; and recommends, that when the patient is not taking that kind of active exercise which invigorates the body, he should lie down—not extended, but in an easy position.

Curvatures of the spine from DISEASE of the VERTEBRÆ are of graver moment; and with a reference to these, we shall close the volume, of which we have given a more extended analysis than we at first designed:—

"The bodies of the vertebræ are of a spongy substance. The ends of the bones are also spongy, and scrofula may arise, and a curvature of the back be produced from a bend forward, and where many of the vertebræ are gone, then one of the vertebræ sticks out, and makes a very acute angle; and this angle, this sharp projection, is one cause of a surgeon's knowing the nature of the disease. Now, in the commencement of the disease, the question is, whether it begins in the bone or in the intervertebral substance? Now, I really cannot tell. Sometimes it is in the one, and sometimes in the other. In all probability, it is very rare that we have an opportunity of examining it at an early period. When scrofula begins, the disease goes on to ulceration, and the bodies of the vertebræ are crushed by weight. It is right to tell you, too, that you may have the disease without curvature, and you may have curvature without the disease." —p. 343.

To treat a case of this kind successfully, rest and a recumbent posture are absolutely necessary; but the measures requisite to a restoration of the general health, are not to be overlooked. Of the issue-practice, recommended by Mr. Pott, and so generally adopted by the profession, Mr. A. says nothing. In the early stages, cupping, with purgatives, is undoubtedly beneficial.

(TO BE CONTINUED.)

